# The tryptophan–kynurenine pathway in immunomodulation and cancer metastasis

**DOI:** 10.1002/cam4.6484

**Published:** 2023-08-29

**Authors:** Charlise Basson, June Cheptoo Serem, Yvette Nkondo Hlophe, Priyesh Bipath

**Affiliations:** ^1^ Department of Physiology, School of Medicine University of Pretoria Pretoria South Africa; ^2^ Department of Anatomy, School of Medicine University of Pretoria Pretoria South Africa

**Keywords:** Ahr, cancer, immunoregulation, kynurenine, metastasis, tryptophan pathway

## Abstract

**Introduction:**

The activation of the kynurenine pathway in cancer progression and metastasis through immunomodulatory pathways has drawn attention to the potential for kynurenine pathway inhibition. The activation of the kynurenine pathway, which results in the production of kynurenine metabolites through the degradation of tryptophan, promotes the development of intrinsically malignant properties in cancer cells while facilitating tumour immune escape. In addition, kynurenine metabolites act as biologically active substances to promote cancer development and metastasis.

**Methods:**

A literature review was conducted to investigate the role of the tryptophan‐kynurenine pathway in immunomodulation and cancer metastasis.

**Results:**

Evidence suggests that several enzymes and metabolites implicated in the kynurenine pathway are overexpressed in various cancers. As such, the tryptophan pathway represents a promising target for cancer treatment. However, downstream signalling pathways, including aryl hydrocarbon receptor activation, have previously induced diverse biological effects in various malignancies, which resulted in either the promotion or the inhibition of metastasis.

**Conclusion:**

As a result, a thorough investigation of the kynurenine pathway and its regulatory mechanisms is necessary in order to properly comprehend the effects of kynurenine pathway activation involved in cancer development and metastasis.

## Graphical abstract

The activation of the kynurenine pathway, which results in the synthesis of kynurenine metabolites through the breakdown of tryptophan, promotes the development of intrinsically malignant properties in cells while encouraging tumour immune escape. Even though the tryptophan pathway represents a promising target for the treatment of cancer, downstream signalling pathways, including aryl hydrocarbon receptor activation, have previously caused diverse biological consequences in various malignancies, which may result in either pro‐ or anti‐metastatic effects. As a result, an in‐depth examination of regulatory mechanisms is necessary in order to properly comprehend the kynurenine pathway and its signalling mechanisms involved in cancer development and metastasis.

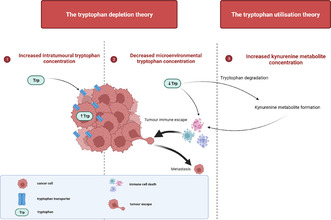

## 1 INTRODUCTION

Cancer metastasis is the primary cause of cancer mortality, accounting for approximately 90% of cancer‐related deaths and remains a challenge in treatment.^1^, ^2^, ^3^, ^4^ This hallmark of cancer refers to the dissemination of cancer cells through a series of sequential steps from the initial tumour to a distant secondary site.^4^ Metastasis is often promoted by immunomodulatory processes leading to tumour cell immune escape and a favourable pre‐metastatic niche.^1^ As such, the increased interest in the possibility of kynurenine pathway inhibition can be attributed to the role of kynurenine metabolites and enzymes in cancer progression and metastasis through immunomodulatory pathways. These pathways promote the progression of the kynurenine pathway, leading to the production of kynurenine metabolites in the kynurenine pathway through tryptophan degradation. Tryptophan degradation, leading to its depletion, contributes to tumour cell immune escape and the advancement of the intrinsic malignant properties of cells,^5^, ^6^ while yielding kynurenine metabolites that act as biologically active substances to promote cancer development through immunomodulatory mechanisms.^7^, ^8^

As a result, the overexpression of the tryptophan‐degrading enzymes such as indoleamine 2,3‐dioxygenase (IDO) and tryptophan 2,3‐dioxygenase (TDO) in cancer has led to extended research on the tryptophan–kynurenine pathway in cancer.^7^, ^9^ The activation of the kynurenine pathway in cancer involves a variety of enzymes, which includes, but is not limited to IDO1 or IDO2, TDO, kynurenine formamidase, kynurenine‐3‐hydroxylase, kynurenine aminotransferase, kynureninase, 3‐hydroxyanthranilic acid oxygenase, quinolinate phosphoribosyltransferase (QPRT), nicotinamide mononucleotide adenylyltransferase (NMNAT) and Kynurenine 3‐monooxygenase (KMO).^7^, ^10^ The kynurenine pathway produces a variety of biologically active metabolites, such as kynurenine, 3‐hydroxykynurenine, 3‐hydroxy‐l‐kynurenamine, kynurenic acid, quinolinic acid, picolinic acid and xanthurenic acid which influence numerous physiological processes and has shown to contribute to carcinogenesis and metastasis.^7^

This review will focus on the mechanisms involved in kynurenine‐mediated cancer progression and metastasis through tryptophan depletion leading to downstream metabolite utilisation, with particular focus on kynurenic acid, quinolinic acid and kynurenine through receptor activation. In addition, several treatment strategies to intervene with kynurenine pathway progression will also be discussed.

## 2 THE KYNURENINE PATHWAY AND DOWNSTREAM METABOLITES

Under physiological conditions, tryptophan is an essential amino acid for maintaining normal cellular functioning, such as protein translation.^11^ Tryptophan can be metabolised through various processes, including decarboxylation to tryptamine, protein synthesis, the serotonin pathway and the kynurenine pathway, where the latter contributes to 95% of all dietary tryptophan consumed by mammals.^12^ However, in cancer, it has been reported that kynurenine pathway activation has been associated with metastasis,^11^ tumour progression and chemoresistance.^13^ As such, an increased kynurenine/tryptophan ratio in the circulation is a well‐known indication of cancer disease progression.^14^, ^15^, ^16^

In the kynurenine pathway, TDO, IDO1 and IDO2 enzyme activity contribute to the rate‐limiting step, responsible for the breakage of the 2,3‐double bond of the indole ring in l‐tryptophan, which leads to N′‐formylkynurenine production.^17^ According to the Human Protein Atlas, TDO2 is highly expressed in the liver and gall bladder,^18^ IDO1 in the respiratory system, gastrointestinal tract, skin, bone marrow and lymphoid tissues,^19^ and high IDO2 RNA expression has been confirmed in liver and gall bladder as well as the female reproductive system.^20^

Additionally, studies suggest that the IDO1 gene (expressed in mammals and fungi) arose from the duplication of IDO2 (expressed in all organisms, including bacteria), and both genes can be found in tandem on chromosome 8 in humans and rodents. Although IDO2 can also activate the kynurenine pathway, its affinity for the tryptophan substrate and its ability to produce kynurenine is very low or almost negligible.^21^ As a result, IDO2 likely plays a minimal role in overall tryptophan metabolism. Despite this, IDO2 is expressed at high levels in some human tumours,^21^, ^22^ such as non‐small cell carcinoma, pancreatic cancers and cervical cancer.^23^ In addition, evidence suggests that IDO1, TDO and IDO2 may all play a role in malignant tumours. However, the three enzymes differ in their expression patterns, regulatory mechanisms and functions within various tumour microenvironments,^24^ but a consensus has not been reached. Therefore, more research on the role of IDO2 is needed to confirm its role in cancer and identify potential therapies to target it.^23^

Overall, IDO has been shown to induce immunosuppression, cancer cell proliferation, invasion and migration^16^, ^25^, ^26^, ^27^ through three main immunomodulatory mechanisms: (1) promoting the formation of immunosuppressive antigen‐presenting cells, (2) increasing tryptophan consumption and kynurenine metabolite production which activates signalling pathways, such as aryl hydrocarbon receptors (Ahr) activation leading to T‐cell apoptosis and the suppression of T‐helper 17 cells (Th17) and (3) inducing myeloid‐derived suppressor cells (MDSCs), which migrate to tumour tissues to produce an immunosuppressive tumour microenvironment.^27^ Higher plasma IDO1 activity has been documented in lung, gynaecological, breast, colorectal and melanoma malignancies, and upregulated expression of IDO in the microenvironment of laryngeal and oesophageal carcinomas.^28^ In cancer, IDO elevation is often caused as a response to inflammation, specifically by other proinflammatory cytokines, including tumour necrosis factor α (TNFα), interleukin‐1 (IL‐1), IL‐6, interferon‐gamma (IFN‐γ), transforming growth factor beta (TGF‐β), cytotoxic T‐lymphocyte‐associated protein 4 (CTLA‐4) and programmed cell death protein 1 (PD‐1).^27^, ^29^

Previously, IDO1‐positive P815 murine mastocytoma clones demonstrated metabolic interactions between the tumour and the microenvironment, which led to cell‐typing analysis that could be distilled into three general states: growth of tumour cells, suppression of effector functions and metabolism of tumour‐associated T cells, and promotion of a tolerogenic microenvironment.^30^ Another study investigating the function of IDO inhibition using a mouse graft‐versus‐tumour model of reduced‐intensity allogeneic haematopoietic stem cell transplant, followed by donor leukocyte infusion found that an IDO inhibitor decreased the growth of the tumour and increased the expression of IDO1 and IFN‐γ in mice that received donor leukocyte infusion.^31^ On the other hand, mice that did not receive donor leukocyte infusion did not express IDO1 and IFN‐γ, implying that IDO inhibition can be advantageous for anti‐tumour therapy when used in conjunction with reduced‐intensity allogeneic haematopoietic stem cell transplant with donor leukocyte infusion.^31^ Patients with nasopharyngeal carcinoma have previously been shown to have higher plasma IDO levels and local expression alterations. Furthermore, elevated plasma IDO levels were previously associated with poorer patient survival rates. Interestingly, compared to patients without metastasis, there was a significant difference in plasma IDO levels between healthy controls and nasopharyngeal carcinoma patients with metastasis.^28^

Similar to IDO1, TDO is expressed in several cancers, including brain, lung and breast cancers, and has been reported to induce immunomodulation while promoting tumour immune resistance and proliferation.^28^, ^32^ In oesophageal squamous cell carcinoma, TDO expression previously correlated with tumour stage, its recurrence and the presence of CD44+ stem cells.

Additionally, TDO inhibition reduced epidermal growth factor (EGF) signalling to inhibit oesophageal squamous cell carcinoma cell proliferation.^33^, ^34^

**FIGURE 1:**
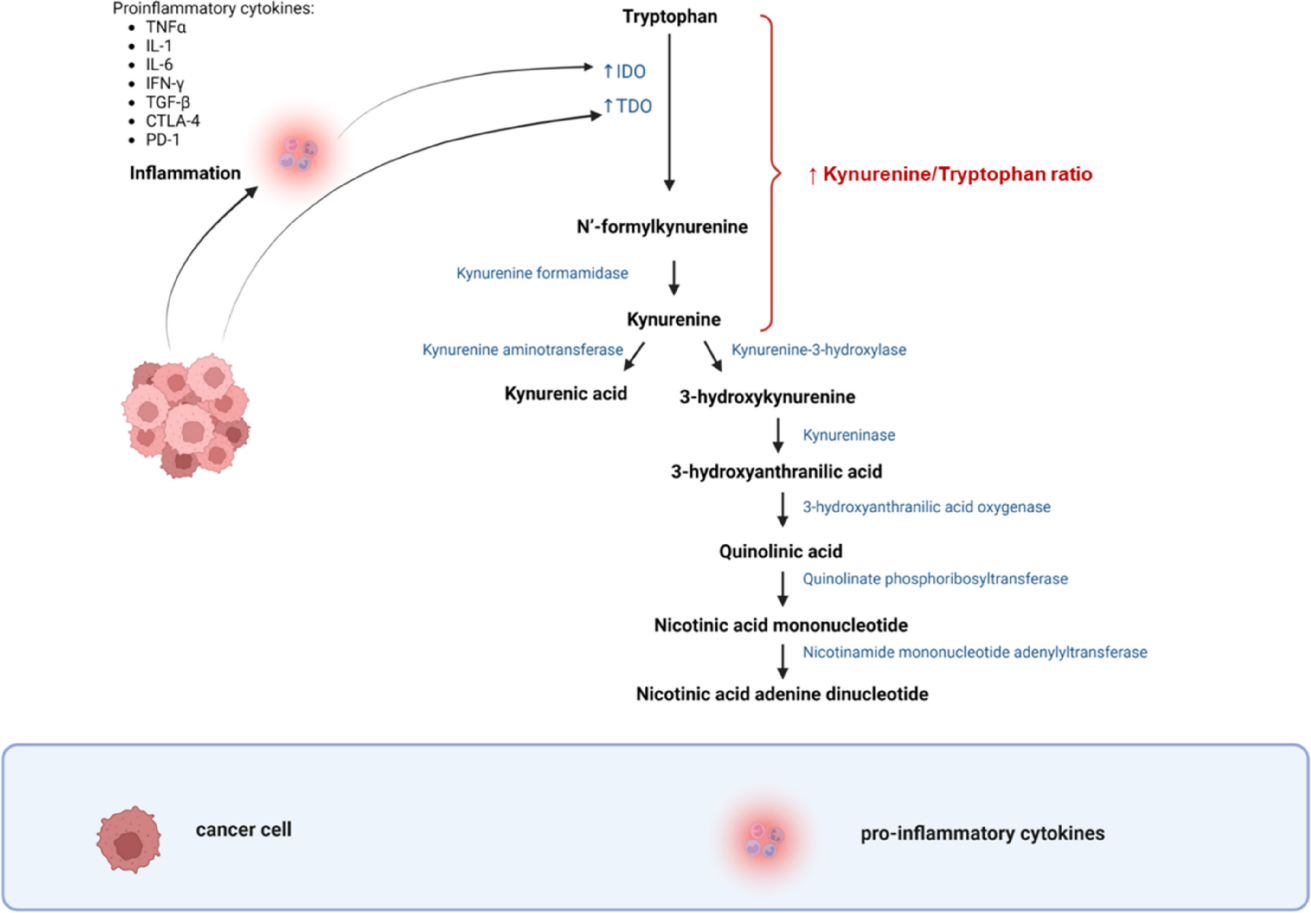
Tryptophan degradation leading to the formation of kynurenine metabolites. Tryptophan degradation is promoted by the cancer‐associated increase in indoleamine IDO/TDO expression, leading to the formation of N′‐formylkynurenine. The latter is hydrolysed to kynurenine by the enzyme kynurenine formamidase. Kynurenine may form kynurenic acid or follow a cascade of enzymatic reactions to produce NAD^+^. CTLA‐4, cytotoxic T‐lymphocyte‐associated protein 4; IDO, indoleamine 2,3‐dioxygenase; IFN‐γ, interferon‐gamma; IL, interleukin; NAD^+^, nicotinamide adenine dinucleotide; PD‐1, programmed cell death protein 1; TDO, tryptophan 2,3‐dioxygenase; TGF‐β, transforming growth factor beta; TNFα, tumour necrosis factor α.^6^, ^11^, ^12^ Image created by Charlise Basson using BioRender (https://biorender.com/).

In the kynurenine pathway, N′‐formylkynurenine is hydrolysed to l‐kynurenine by kynurenine formamidase.^12^ From kynurenine, the kynurenine pathway branches into various pathways to yield several metabolites (Figure 1).^6^ While limited studies have been conducted on kynurenine metabolites in cancer, these metabolites are thought to have immunomodulatory effects.^11^ The central metabolite, namely kynurenine, is thought to be exported into the tumour microenvironment, contributing to immune suppression, T‐cell evasion and cancer cell survival.^35^ However, when tested directly on melanoma cells in vitro, kynurenine inhibited deoxyribonucleic acid (DNA) synthesis and caused necrosis.^36^

One of the end metabolites in the kynurenine pathway, namely kynurenic acid, is an agonist of GPR35, which is a receptor that is mainly expressed on immune cells and the gastrointestinal tract.^37^ The kynurenic acid–GPR35 interaction may modify the immunological response to initiate carcinogenesis. In addition, GPR35 signalling via extracellular signal‐regulated kinase (ERK) is implicated in several cellular activities, including proliferation, cell survival and metastasis.^38^ Other kynurenine metabolites, including 3‐hydroxykynurenine (3‐HK), 3‐hydroxyanthranilic acid (3‐HAA) and quinolinic acid, previously induced apoptosis in T cells.^39^, ^40^, ^41^ In addition to its direct effects on immune cells, quinolinic acid also serves as a substrate for (nicotinamide adenine dinucleotide) NAD^+^,^6^ which may also control T‐cell immunity.^42^

## 3 NAD^+^ PATHWAYS IN CANCER METASTASIS

NAD^+^ is an essential molecule implicated in cellular function, survival, repair and metabolism (by serving as an electron acceptor for glycolysis, the TCA cycle and fatty acid oxidation).^13^, ^43^ Compared to normal cells, cancer cells typically have elevated NAD^+^ levels to support the cells' higher glycolytic demands necessary for uncontrolled proliferation. NAD^+^ can be produced by three distinct processes, including the salvage pathway, the Preiss–Handler pathway and the de novo biosynthesis pathway through activating the kynurenine pathway.

In cancer cells, NAD^+^ is mainly produced through the salvage pathway, which involves the conversion of nicotinamide and alpha‐d‐5‐phosphoribosyl‐1‐pyrophosphate to nicotinamide mononucleotide (NMN) and subsequently to NAD^+^, which is particularly vital in cancer biology.^44^ Importantly, tumours inhibit NAD^+^ synthesis from tryptophan by host but increase their own NAD^+^ by activating the salvage pathway from nicotinamide.^5^ Many cancer cells, therefore, demonstrate elevated kynurenine enzymes, which contributes to an increase in their own NAD^+^ production.^11^

One of these enzymes, namely QPRT, is highly upregulated in various cancers, including breast, thyroid, colon, ovarian, cervical carcinomas, melanomas, gliomas and lymphomas and its expression is linked to chemoresistance, elevated NAD^+^ production, the activation of purinergic receptors, phosphorylation of the myosin light chain, leading to elongated cell morphology and metastatic features, such as migration and invasion.^13^ In addition, QPRT may interact with caspase‐3, leading to its inactivation and thereby preventing cell death from occurring.^7^

Furthermore, nicotinamide phosphoribosyltransferase (NMPRT), which is the rate‐limiting enzyme in the salvage pathway, shows high expression in various cancer types and its expression levels are inversely correlated with patient survival. Previous studies using NMPRT inhibitors have obtained promising results in preclinical models but not in patients. Another enzyme, namely NMNAT, is upregulated in certain cancers, and its involvement in promoting cell proliferation has been demonstrated.^7^

The reduced form of NAD^+^ is NADH, which serves as an electron donor during oxidative phosphorylation (OXPHOS).^13^ In healthy cells, OXPHOS converts NADH into NAD^+^.^45^ However, cancer cells undergo metabolic reprogramming by switching from glucose metabolism to aerobic glycolysis to sustain uncontrolled proliferation.^45^ Due to OXPHOS being decreased in cancer cells, the conversion of NAD^+^ to NADH is insufficient, which causes lactate dehydrogenase A to produce NADH, allowing for a higher NADH/NAD^+^ redox ratio. A higher NADH/NAD^+^ redox ratio promotes tumour growth and has previously been associated with increased aggressiveness of human breast cancer cells.^45^ Furthermore, the production of NAD^+^ generated from tryptophan degradation previously led to resistance to oxidative stress induced by radiochemotherapy in human gliomas. Therefore, the inhibition of tryptophan degradation may also prevent treatment resistance through the inhibition of NAD^+^ production. However, these effects may be cell line or tissue‐specific as the level of kynurenine metabolite expression may differ in different organs.^46^

## 4 TRYPTOPHAN DEPLETION/UTILISATION THEORY IN CANCER

Despite its physiological function, tryptophan is also a requirement for tumours to support their rapid development and proliferation.^47^ Malignant tissues from cancer patients demonstrated 2.3‐ and 1.5‐times higher tryptophan concentration levels in the stomach and colon, respectively. In addition, tumour tissue in a mouse model with CT26 colon carcinoma demonstrated 2‐times higher tryptophan concentration levels compared to plasma.^47^

Increased intra‐tumoural tryptophan concentrations may be attributed to increased tryptophan absorption caused by elevated tryptophan transporter expression in tumour cells, which eventually leads to tryptophan depletion in the tumour microenvironment. The upregulation of tryptophan transporters SLC1A5 and SLC7A5 is primarily linked to enzymes, including TDO2, FAMID, KAT1, IDO2 and IDO1.^7^ Some studies suggest that the stimulus for this transporter expression is the tryptophan depletion caused by IDO1 or IDO2. However, there is evidence indicating that the Ahr can upregulate the SLC7A5 transporter in response to IDO induction, creating a positive feed‐forward mechanism for Ahr activity.^7^ As a result, immune cells in the tumour microenvironment may perish as a result of this decrease in tryptophan availability.^11^

**FIGURE 2:**
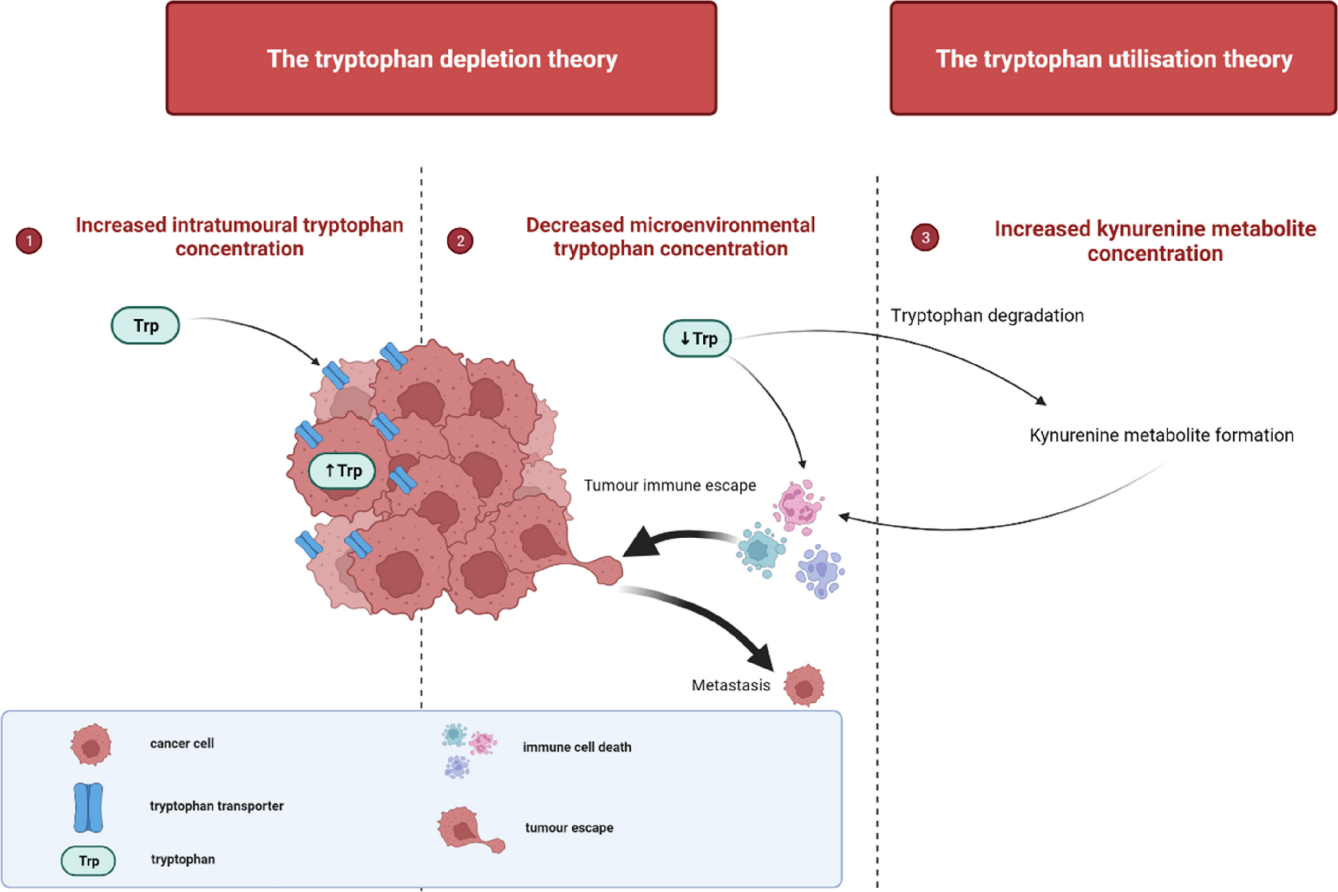
The tryptophan depletion/utilisation theory in cancer. (1) Elevated tryptophan transporter expression in tumour cells leads to increased tryptophan absorption, resulting in increased intra‐tumoural tryptophan concentrations. (2) Tryptophan degradation by IDO/TDO enzymes leads to the formation of kynurenine metabolites. (3) As a result, microenvironmental tryptophan concentrations decrease. Both (2) and (3) may promote immune cell death and result in tumour immune escape, which might lead to metastasis.^11^, ^41^, ^48^, ^49^, ^50^, ^51^, ^52^ Image created by Charlise Basson using BioRender (https://biorender.com/).

Another study demonstrated that tumours induce tryptophan depletion to an unfavourable level for T‐cell survival (lower than 10 μM).^7^ Furthermore, metastasis, proliferation and invasion have previously been upregulated by IDO1.^27^ The tryptophan depletion theory has been substantiated in vitro when T‐cell proliferation was inhibited and apoptosis induced upon the IDO‐mediated removal of tryptophan from the culture medium.^7^ Various studies have associated T‐cell exhaustion with metastasis (Figure 2).^11^, ^48^, ^49^

In addition to the tryptophan depletion theory, the tryptophan utilisation theory explains that the kynurenine metabolites produced from tryptophan degradation in the kynurenine pathway might act as active substances to induce biological effects. Previous studies reported that many of these metabolites also induce immune cell death by inhibiting T‐cell proliferation and promoting apoptosis.^41^, ^50^, ^51^, ^52^

### 4.1 Receptor involvement in cancer

The receptor involvement of kynurenine metabolites has been investigated in various cancers, including melanoma, renal cell carcinoma, colon adenocarcinoma, liver hepatocellular carcinoma and colorectal cancer (Table 1).

**TABLE 1:** The receptor involvement and biological effects of kynurenine metabolites in various cancers.

Type of cancer	Kynurenine metabolites or enzymes involved	Signalling molecule	Biological effect	References
Melanoma (A375)	Kynurenine	Ahr	Inhibited DNA synthesis, induced necrosis but not apoptosis	^36^
	6‐formylindolo[3,2‐b]carbazole (FICZ)		Inhibited DNA synthesis, induced apoptosis, and necrosis	
	Kynurenic acid		Did not inhibit DNA synthesis, induced apoptosis, and necrosis	
Human liver hepatocellular carcinoma (HepG2)	Kynurenic acid Xanthurenic acid	Ahr	Activation of immune escape surveillance mechanisms through the elevation of IL‐6 expression	^53^
Human colon adenocarcinoma HT‐29	Kynurenic acid	p21 Waf1/Cip1	Inhibition of proliferation and DNA synthesis	^54^
Colorectal cancer cells (HT‐29 and LS‐180 cells)	8‐Hydroxyquinaldic Acid	Cell line–dependent changes in cell cycle regulator proteins (CDK4, CDK6, cyclin D1, cyclin E) and CDKs inhibitors (p21 Waf1/Cip1, p27 Kip1)	Inhibition of proliferation and migration	^55^
Human renal cell carcinoma Caki‐2	Kynurenic acid	Overexpression of p21 Waf1/Cip1, inhibition of phosphorylation of Rb protein and p38 MAPK	Inhibition of proliferation, DNA synthesis and migration	^56^
Human colon adenocarcinoma HT‐29	Kynurenic acid	ERK1/2, AKT	Inhibition of colon cancer cell proliferation	^57^

**FIGURE 3:**
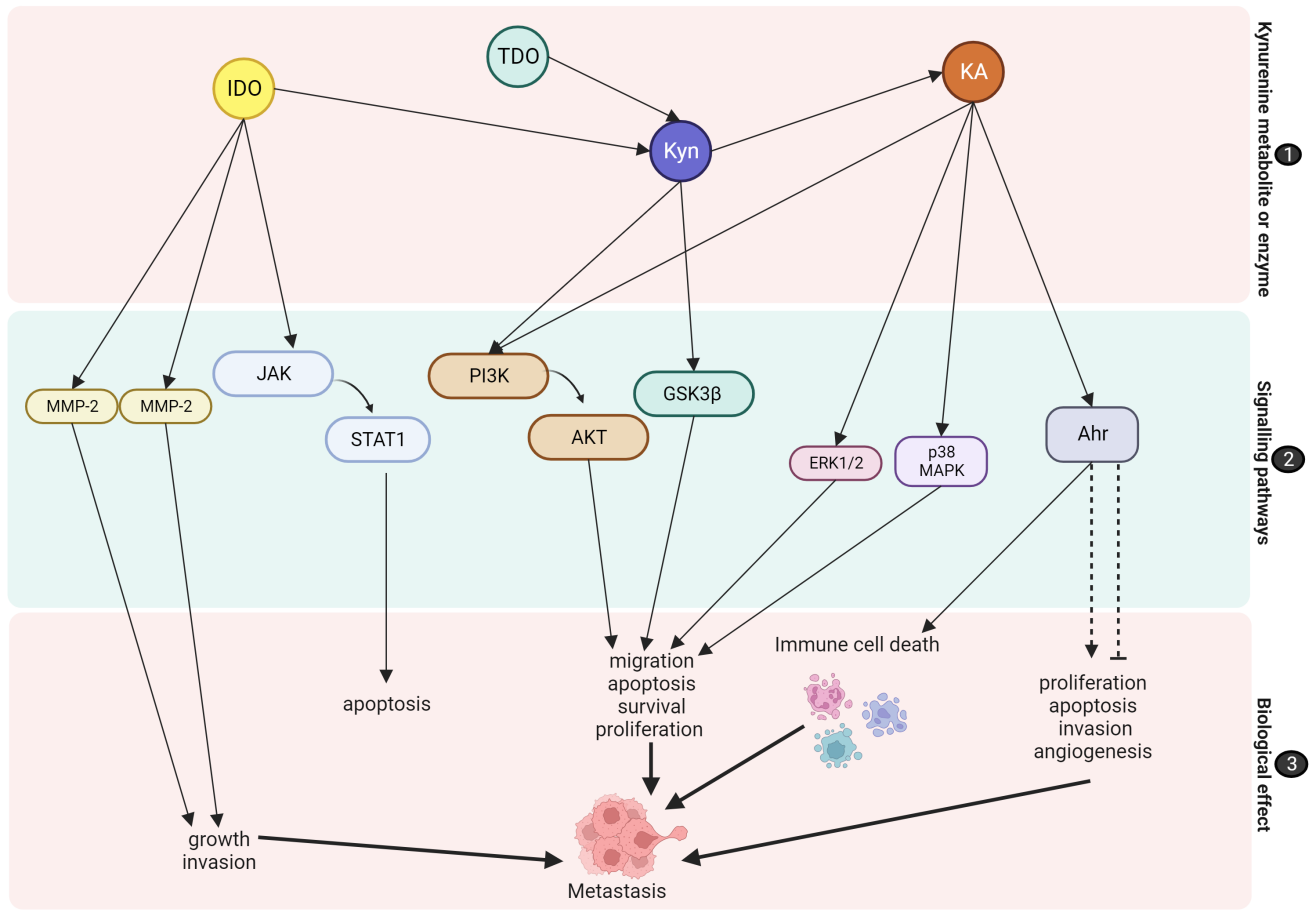
Receptor signalling in the kynurenine pathway leading to metastasis. (1) Kynurenine metabolites and enzymes activate (2) signalling pathways, such as JAK‐STAT1, MMP‐1 and MMP‐2, PI3K‐AKT, GSK3β, ERK1/2, p38 MAPK and Ahr. The activation of these signalling pathways induces (3) biological effects, which may promote metastasis. Dotted lines resemble pathways that may induce contrasting effects as consensus has not been reached. Ahr, aryl hydrocarbon receptors; AKT, protein kinase B; ERK, extracellular signal‐regulated kinase; IDO, indoleamine 2,3‐dioxygenase; JAK, Janus kinas; KA, kynurenic acid; Kyn, kynurenine, MAPK, mitogen‐activated protein kinase; MMP, matrix metalloproteinase; PI3K, phosphoinositide 3‐kinase; STAT, signal transducer and activator of transcription.^13^, ^34^, ^38^, ^52^, ^59^, ^62^ Image created by Charlise Basson using BioRender (https://biorender.com/).

IDO has been reported to promote adhesion, invasion, metastasis and angiogenesis of vascular endothelial cells but demonstrated limited effects on the proliferation of Lewis lung cancer cells. In addition, IDO overexpression increased matrix metalloproteinase‐2 (MMP‐2) and MMP‐9.^58^ Matrix metalloproteinases are implicated in cancer development and metastasis through promoting tumour growth and invasion. Moreover, IDO activation by IFN‐γ activates the JAK‐STAT1 pathway, resulting in apoptosis (Figure 3). In mixed lymphocyte reactions (MLRs), IDO inhibition decreased the expression of p53 and p21 in T cells and led to apoptosis.^59^ The pro‐apoptotic effects of IDO (through caspase‐3 and ‐9 activation) have also been reported.^59^, ^60^

Furthermore, the kynurenine pathway has been reported to activate many signalling pathways, including ERK, phospoinositide‐3 kinase (PI3K), Wnt/−catenin, P53, bridging integrator 1 (BIN1), cyclooxygenase‐2 (COX‐2), cyclin‐dependent kinase (CDK) and collagen type XII alpha 1 chain (COL12A1). Another study found that cell proliferation was associated with kynurenine‐mediated activation of the PI3K‐AKT‐GSK3β signalling pathways, which induce β‐catenin stabilisation.^13^ These signalling pathways are involved in cellular processes, including migration, apoptosis, survival, proliferation and metastasis (Figure 3).^34^, ^38^

In addition, kynurenine metabolites, including kynurenine and kynurenic acid, may activate Ahr.^52^ A previous study by DiNatale et al. demonstrated that kynurenine exhibited minimal Ahr agonist activity, while KA exhibited the highest potency, followed by xanthurenic acid.^53^ Both cancer patients and tumour‐bearing animals have demonstrated the existence of the activated Ahr pathway, and it has been proposed that blocking this pathway can improve the efficacy of anticancer adoptive T‐cell treatment.^61^ As previously discussed, Ahr signalling has been associated with immune evasion (Figure 3). Mounting evidence suggests that immunosuppression, contributing to immune evasion, is conducive to tumour progression. As such immune evasion has been identified as an emerging hallmark of cancer. In addition to its immunosuppressive effects, Ahr may directly promote cell proliferation, apoptosis, invasion, angiogenesis and metastasis.^38^, ^62^ Yin et al. described that Ahr may promote cell survival and proliferation through a variety of mechanisms, which include the modulation of receptor expression, its involvement in growth factor signalling, its anti‐apoptotic effects, regulation of cell cycle and ability to promote cell cytokine expression.^63^ Additionally, kynurenine activates Ahr, which leads to in apoptosis, adhesion, migration, immune cell differentiation, tumour suppression, cancer promotion and cell proliferation.^64^ The hypothesis that the kynurenine pathway promotes Ahr activity in cancer cells is supported by the previous observation that Ahr is constitutively active in tryptophan‐catabolising cancer cells. In addition, the inhibition of tryptophan catabolism either by deletion of TDO and/or IDO1 or IDO2 or by tryptophan depletion leads to suppression of constitutive Ahr activity.^65^

However, contrasting effects on Ahr signalling have been reported, which may resemble a double‐edged sword (Figure 3).^52^ While inhibiting neuroblastoma progression and metastasis, Ahr activation promoted tumour growth, migration and metastasis in hepatocellular carcinoma.^7^ Furthermore, Ahr agonists previously induced MMPs and led to invasion and migration in melanoma cells (A2058).^66^ Platten et al. reported that kynurenine‐mediated Ahr activation has a pro‐tumourigenic effect on tumour cells, increasing cell motility and clonogenic survival.^65^ Furthermore, a study on ovarian cancer showed that IDO1‐induced kynurenine production stimulated Ahr nuclear translocation, leading to T‐cell malfunction. This study suggested that strategies targeting IDO1 and Ahr pathways may be able to override immune repression.^67^ In addition to its effect on metastasis, other studies found that the tryptophan‐kynurenine‐Ahr signalling pathway increased stemness in oral tongue squamous cell carcinomas cells, which promoted tumour dormancy,^68^ while promoting survival and drug resistance in leukaemic cells.^69^ However, Menacho‐Márquez et al. previously demonstrated the contrasting effects of Ahr in B16 F10 melanoma cells, showing that Ahr contributes to the tumour–stroma interaction and inhibits cell growth and metastasis when expressed in the tumour cell but induces these effects when expressed in the stroma.^70^ Therefore, more research is necessary to fully elucidate its role in carcinogenesis and cancer metastasis.

Despite its effect on the activation of Ahr, kynurenic acid may also regulate G‐protein receptor‐mediated signalling pathways, including the PI3K/protein kinase B (Akt) and mitogen‐activated protein kinase (MAPK) pathways. Kynurenic acid has been shown to inhibit cancer‐associated ERK 1/2, p38 MAPK and Akt, which play a direct role in the cellular processes, such as migration, survival, proliferation and apoptosis (Figure 3).^38^ When compared to kynurenic acid and FICZ, l‐kynurenine displayed the most potent anti‐proliferative effects on normal human adult primary epidermal melanocytes (HEMa) and human melanoma A375 and RPMI7951.^36^ However, other studies have demonstrated the pro‐carcinogenic properties of KA, as demonstrated by the enhanced proliferation of mouse microglia and human glioblastoma cells, in the presence of KA.^71^

## 5 THERAPIES INVOLVING THE KYNURENINE PATHWAY FOR CANCER TREATMENT

Therapies involving the kynurenine pathway for cancer treatment have been investigated in various cancers. IDO/TDO inhibition and Ahr modulation will be discussed below.

### 5.1 IDO/TDO inhibition

IDO1 has emerged as an attractive target for cancer treatment due to its involvement in mediating cancer immune tolerance and its relationship with a poor cancer prognosis.^72^ Furthermore, numerous reviews have recently discussed the effects of IDO inhibitors in detail.^34^, ^50^ The IDO inhibitors, namely epacadostat, indoximod, BMS986205, novaximod (GDC‐0919) and PF‐0684003, have advanced to 97 different clinical trials for cancer treatment.^72^ In patients with advanced malignancies, the first‐generation IDO1 inhibitors, such as indoximod and epacadostat, have not indicated significant antitumour effects when used as monotherapy. However, when combining epacadostat with the PD‐1 immune checkpoint inhibitor pembrolizumab, long‐lasting effects were observed. Interestingly, the combination of navoximod and the PD‐L1 antibody atezolizumab failed to elicit significant results. In addition, cancers including non‐small cell lung cancer, renal cell carcinoma, squamous cell carcinoma of the head and neck, and melanoma demonstrated more significant clinical outcomes in studies involving IDO1 inhibitors and checkpoint inhibitors. These drug combinations are still being investigated in clinical trials.^73^

However, IDO1 inhibition may not completely inhibit the kynurenine pathway activation as both IDO1 and TDO are overexpressed in many cancers and responsible for the catabolism of tryptophan. Therefore, dual IDO1 and TDO inhibitors are currently being investigated in a physical trial (NCT03491631). In addition, another clinical has been conducted on dual IDO1 and TDO inhibitors in solid tumours (NCT03208959). However, the results have not been made available.^73^

### 5.2 Ahr modulation

Ahr ligands (agonists) include FICZ and kynurenine, bilirubin, indigoids and chemoprotective phytochemicals, such as flavonoids and indole‐3‐carbinol.^66^ By directly inducing immunosuppression, Ahr activation is widely acknowledged to generate an immunological tolerance response. Therefore, inhibiting Ahr activation through synthetic antagonists is an alternate method to reroute immunity toward tumour rejection, in addition to preventing the synthesis of kynurenine metabolites.^73^ However, a major challenge in terms of Ahr inhibition is promiscuous ligand binding.^52^ Ahr antagonists have been developed and include CH‐223191, StemRegenin 1 (SR1) and CB7993113.^46^ However, these compounds have not been studied in cancer.^73^

## 6 CONCLUSION

The activation of the kynurenine pathway and the production of kynurenine metabolites produced by tryptophan degradation play an essential role in cancer development and metastasis through immunomodulatory pathways. Due to the immunosuppressive and potential pro‐metastatic effects of tryptophan‐associated enzymes and kynurenine metabolites, the tryptophan pathway represents a promising target for treating cancer. However, downstream signalling pathways, such as Ahr activation, previously induced contrasting biological effects in different cancers, rendering Ahr signalling pro‐metastatic as well as anti‐metastatic. Even though various drugs and combination therapies altering the kynurenine pathway are currently being investigated in clinical trials, additional research is needed to fully elucidate the effects of these inhibitors on various types of cancers. Therefore, the kynurenine pathway and its signalling mechanisms demand a proper understanding. Furthermore, developing combined approaches to target the kynurenine pathway along with other aberrant cancer‐associated signalling pathways may represent a promising strategy to manage the metastatic burden.

## AUTHOR CONTRIBUTIONS

**Charlise Basson:** Conceptualization (equal); writing – original draft (lead); writing – review and editing (equal). **June Cheptoo Serem:** Conceptualization (equal); writing – review and editing (equal). **Yvette Nkondo Hlophe:** Conceptualization (equal); writing – review and editing (equal). **Priyesh Bipath:** Conceptualization (equal); writing – review and editing (equal).

## FUNDING INFORMATION

The author(s) disclosed receipt of the following financial support for the research, authorship and/or publication of this article: The Research Development program of Dr YN Hlophe and Dr JC Serem by the University of Pretoria. School of Medicine Research Committee (RESCOM) Grant awarded to Ms. Basson. National Research Foundation (NRF) awarded to Prof R Anguelov and Dr Hlophe.

## CONFLICT OF INTEREST STATEMENT

The author(s) declared no potential conflicts of interest with respect to the research, authorship and/or publication of this article.

## ETHICS STATEMENT

The ethical consent for this study was obtained from the University of Pretoria, Faculty of Health Science, Research Ethics Committee (reference number: 405/2020).

## Data Availability

Data sharing not applicable to this article as no datasets were generated or analysed during the current study.

## References

[1] Guan X . Cancer metastases: challenges and opportunities. Acta Pharm Sin B. 2015;5(5):402‐418. 26579471 10.1016/j.apsb.2015.07.005 PMC4629446

[2] Mehlen P , Puisieux A . Metastasis: a question of life or death. Nat Rev Cancer. 2006;6(6):449‐458. 16723991 10.1038/nrc1886

[3] Seyfried TN , Huysentruyt LC . On the origin of cancer metastasis. Crit Rev Oncog. 2013;18(1–2):43‐73. 23237552 10.1615/critrevoncog.v18.i1-2.40 PMC3597235

[4] Fares J , Fares MY , Khachfe HH , Salhab HA , Fares Y . Molecular principles of metastasis: a hallmark of cancer revisited. Signal Transduct Target Ther. 2020;5(1):28. 32296047 10.1038/s41392-020-0134-x PMC7067809

[5] Navas LE , Carnero A . NAD^+^ metabolism, stemness, the immune response, and cancer. Signal Transduct Target Ther. 2021;6(1):2. 33384409 10.1038/s41392-020-00354-w PMC7775471

[6] Bello C , Heinisch PP , Mihalj M , Carrel T , Luedi MM . Indoleamine‐2,3‐dioxygenase as a perioperative marker of the immune system. Front Physiol. 2021;12:766511. 34819875 10.3389/fphys.2021.766511 PMC8606526

[7] Badawy AA . Tryptophan metabolism and disposition in cancer biology and immunotherapy. Biosci Rep. 2022;42(11):BSR20221682. 36286592 10.1042/BSR20221682 PMC9653095

[8] Adamo A , Frusteri C , Pallotta MT , Pirali T , Sartoris S , Ugel S . Moonlighting proteins are important players in cancer immunology. Front Immunol. 2021;11:613069. 33584695 10.3389/fimmu.2020.613069 PMC7873856

[9] Badawy AA . Kynurenine pathway of tryptophan metabolism: regulatory and functional aspects. Int J Tryptophan Res. 2017;10:1178646917691938. 28469468 10.1177/1178646917691938 PMC5398323

[10] Chen Y , Zhang J , Yang Y , et al. Kynurenine‐3‐monooxygenase (KMO): from its biological functions to therapeutic effect in diseases progression. J Cell Physiol. 2022;237(12):4339‐4355. 36088660 10.1002/jcp.30876

[11] Perez‐Castro L , Garcia R , Venkateswaran N , Barnes S , Conacci‐Sorrell M . Tryptophan and its metabolites in normal physiology and cancer etiology. FEBS J. 2023;290(1):7‐27. 34687129 10.1111/febs.16245 PMC9883803

[12] Austin CJ , Rendina LM . Targeting key dioxygenases in tryptophan–kynurenine metabolism for immunomodulation and cancer chemotherapy. Drug Discov Today. 2015;20(5):609‐617. 25478733 10.1016/j.drudis.2014.11.007

[13] Gouasmi R , Ferraro‐Peyret C , Nancey S , et al. The kynurenine pathway and cancer: why keep it simple when you can make it complicated. Cancers (Basel). 2022;14(11):2793. 35681770 10.3390/cancers14112793 PMC9179486

[14] Suzuki Y , Suda T , Furuhashi K , et al. Increased serum kynurenine/tryptophan ratio correlates with disease progression in lung cancer. Lung Cancer. 2010;67(3):361‐365. 19487045 10.1016/j.lungcan.2009.05.001

[15] Meireson A , Ferdinande L , Haspeslagh M , et al. Clinical relevance of serum kyn/trp ratio and basal and ifnγ‐upregulated ido1 expression in peripheral monocytes in early stage melanoma. Front Immunol. 2021;12:736498. 34557196 10.3389/fimmu.2021.736498 PMC8453201

[16] Badawy AA‐B , Guillemin G . The plasma [kynurenine]/[tryptophan] ratio and indoleamine 2,3‐dioxygenase: time for appraisal. IJTR. 2019;12:1178646919868978. 31488951 10.1177/1178646919868978 PMC6710706

[17] Zhai L , Ladomersky E , Bell A , et al. Quantification of IDO1 enzyme activity in normal and malignant tissues. Methods Enzymol. 2019;629:235‐256. 31727243 10.1016/bs.mie.2019.07.006 PMC7347075

[18] TDO2 . The human protein atlas. Accessed August 8, 2013. https://www.proteinatlas.org/ENSG00000151790‐TDO2

[19] IDO1 . The human protein atlas. Accessed August 8, 2013. https://www.proteinatlas.org/ENSG00000131203‐IDO1/tissue

[20] IDO2 . The human protein atlas. Accessed August 8, 2013. https://www.proteinatlas.org/ENSG00000188676‐IDO2/tissue

[21] Mondanelli G , Mandarano M , Belladonna ML , et al. Current challenges for IDO2 as target in cancer immunotherapy. Front Immunol. 2021;12:679953. 33968089 10.3389/fimmu.2021.679953 PMC8097162

[22] Zhai L , Ladomersky E , Lenzen A , et al. IDO1 in cancer: a gemini of immune checkpoints. Cell Mol Immunol. 2018;15(5):447‐457. 29375124 10.1038/cmi.2017.143 PMC6068130

[23] Li P , Xu W , Liu F , et al. The emerging roles of IDO2 in cancer and its potential as a therapeutic target. Biomed Pharmacother. 2021;137:111295. 33550042 10.1016/j.biopha.2021.111295

[24] Song X , Si Q , Qi R , et al. Indoleamine 2,3‐dioxygenase 1: a promising therapeutic target in malignant tumor. Front Immunol. 2021;12:800630. 35003126 10.3389/fimmu.2021.800630 PMC8733291

[25] Xiang Z , Li J , Song S , et al. A positive feedback between IDO1 metabolite and COL12A1 via MAPK pathway to promote gastric cancer metastasis. J Exp Clin Cancer Res. 2019;38(1):314. 31315643 10.1186/s13046-019-1318-5 PMC6637527

[26] Xu M , Zhu F , Yin Q , et al. Serum response factor‐regulated IDO1/Kyn‐Ahr pathway promotes tumorigenesis of oral squamous cell carcinoma. Cancers (Basel). 2023;15(4):1319. 36831659 10.3390/cancers15041319 PMC9954402

[27] Ye Z , Yue L , Shi J , Shao M , Wu T . Role of IDO and TDO in cancers and related diseases and the therapeutic implications. J Cancer. 2019;10(12):2771‐2782. 31258785 10.7150/jca.31727 PMC6584917

[28] Boros FA , Vécsei L . Immunomodulatory effects of genetic alterations affecting the kynurenine pathway. Front Immunol. 2019;10:2570. 31781097 10.3389/fimmu.2019.02570 PMC6851023

[29] Meireson A , Devos M , Brochez L . Ido expression in cancer: different compartment, different functionality? Front Immunol. 2020;11:531491. 33072086 10.3389/fimmu.2020.531491 PMC7541907

[30] Ait‐Belkacem R , Bol V , Hamm G , et al. Microenvironment tumor metabolic interactions highlighted by qmsi: application to the tryptophan–kynurenine pathway in immuno‐oncology. SLAS Discov. 2017;22(10):1182‐1192. 28557618 10.1177/2472555217712659

[31] Lim JY , Lee SE , Park G , Choi EY , Min CK . Inhibition of indoleamine 2,3‐dioxygenase by stereoisomers of 1‐methyl tryptophan in an experimental graft‐versus‐tumor model. Exp Hematol. 2014;42(10):862‐866.e3. 24971697 10.1016/j.exphem.2014.06.006

[32] Zhai L , Bell A , Ladomersky E , et al. Immunosuppressive IDO in cancer: mechanisms of action, animal models, and targeting strategies. Front Immunol. 2020;11:1185. 32612606 10.3389/fimmu.2020.01185 PMC7308527

[33] Pham QT , Oue N , Sekino Y , et al. TDO2 overexpression is associated with cancer stem cells and poor prognosis in esophageal squamous cell carcinoma. Oncology. 2018;95(5):297‐308. 30134247 10.1159/000490725

[34] Ala M . The footprint of kynurenine pathway in every cancer: a new target for chemotherapy. Eur J Pharmacol. 2021;896:173921. 33529725 10.1016/j.ejphar.2021.173921

[35] Venkateswaran N , Conacci‐Sorrell M . Kynurenine: an oncometabolite in colon cancer. Cell Stress. 2020;4(1):24‐26. 31922097 10.15698/cst2020.01.210 PMC6946015

[36] Walczak K , Langner E , Makuch‐Kocka A , et al. Effect of tryptophan‐derived AhR ligands, kynurenine, kynurenic acid and FICZ, on proliferation, cell cycle regulation and cell death of melanoma cells‐in vitro studies. Int J Mol Sci. 2020;21(21):7946. 33114713 10.3390/ijms21217946 PMC7663343

[37] Wang J , Simonavicius N , Wu X , et al. Kynurenic acid as a ligand for orphan G protein‐coupled receptor GPR35. J Biol Chem. 2006;281(31):22021‐22028. 16754668 10.1074/jbc.M603503200

[38] Walczak K , Wnorowski A , Turski WA , Plech T . Kynurenic acid and cancer: facts and controversies. Cell Mol Life Sci. 2020;77(8):1531‐1550. 31659416 10.1007/s00018-019-03332-w PMC7162828

[39] Hayashi T , Mo JH , Gong X , et al. 3‐Hydroxyanthranilic acid inhibits PDK1 activation and suppresses experimental asthma by inducing T cell apoptosis. Proc Natl Acad Sci U S A. 2007;104(47):18619‐18624. 18003900 10.1073/pnas.0709261104 PMC2141826

[40] Mailankot M , Nagaraj RH . Induction of indoleamine 2,3‐dioxygenase by interferon‐gamma in human lens epithelial cells: apoptosis through the formation of 3‐hydroxykynurenine. Int J Biochem Cell Biol. 2010;42(9):1446‐1454. 20435158 10.1016/j.biocel.2010.04.014 PMC2910246

[41] Fallarino F , Grohmann U , Vacca C , et al. T cell apoptosis by tryptophan catabolism. Cell Death Differ. 2002;9(10):1069‐1077. 12232795 10.1038/sj.cdd.4401073

[42] Baumgartner R , Forteza MJ , Ketelhuth DFJ . The interplay between cytokines and the kynurenine pathway in inflammation and atherosclerosis. Cytokine. 2019;122:154148. 28899580 10.1016/j.cyto.2017.09.004

[43] Covarrubias AJ , Perrone R , Grozio A , Verdin E . NAD^+^ metabolism and its roles in cellular processes during ageing. Nat Rev Mol Cell Biol. 2021;22(2):119‐141. 33353981 10.1038/s41580-020-00313-x PMC7963035

[44] Kennedy BE , Sharif T , Martell E , et al. NAD^+^ salvage pathway in cancer metabolism and therapy. Pharmacol Res. 2016;114:274‐283. 27816507 10.1016/j.phrs.2016.10.027

[45] Xie N , Zhang L , Gao W , et al. NAD^+^ metabolism: pathophysiologic mechanisms and therapeutic potential. Signal Transduct Target Ther. 2020;5(1):227. 33028824 10.1038/s41392-020-00311-7 PMC7539288

[46] Platten M , Nollen EAA , Röhrig UF , Fallarino F , Opitz CA . Tryptophan metabolism as a common therapeutic target in cancer, neurodegeneration and beyond. Nat Rev Drug Discov. 2019;18(5):379‐401. 30760888 10.1038/s41573-019-0016-5

[47] Badawy AA . Targeting tryptophan availability to tumors: the answer to immune escape? Immunol Cell Biol. 2018;96(10):1026‐1034. 29888434 10.1111/imcb.12168

[48] Li K , Li T , Feng Z , et al. CD8^+^ T cell immunity blocks the metastasis of carcinogen‐exposed breast cancer. Sci Adv. 2021;7(25):eabd8936. 34144976 10.1126/sciadv.abd8936 PMC8213232

[49] Joseph R , Soundararajan R , Vasaikar S , et al. CD8^+^ T cells inhibit metastasis and CXCL4 regulates its function. BJC. 2021;125(2):176‐189. 33795809 10.1038/s41416-021-01338-5 PMC8292398

[50] Löb S , Königsrainer A , Rammensee H‐G , Opelz G , Terness P . Can we see the wood for the trees? Nat Rev Cancer. 2009;9(6):445‐452. 19461669 10.1038/nrc2639

[51] Frumento G , Rotondo R , Tonetti M , Damonte G , Benatti U , Ferrara GB . Tryptophan‐derived catabolites are responsible for inhibition of T and natural killer cell proliferation induced by indoleamine 2,3‐dioxygenase. J Exp Med. 2002;196(4):459‐468. 12186838 10.1084/jem.20020121 PMC2196046

[52] Nkandeu DS , Basson C , Joubert AM , et al. The involvement of a chemokine receptor antagonist CTCE‐9908 and kynurenine metabolites in cancer development. Cell Biochem Funct. 2022;40(6):608‐622. 35789495 10.1002/cbf.3731

[53] DiNatale BC , Murray IA , Schroeder JC , et al. Kynurenic acid is a potent endogenous aryl hydrocarbon receptor ligand that synergistically induces interleukin‐6 in the presence of inflammatory signaling. Toxicol Sci. 2010;115(1):89‐97. 20106948 10.1093/toxsci/kfq024 PMC2855350

[54] Walczak K , Turski WA , Rzeski W . Kynurenic acid enhances expression of p21 Waf1/Cip1 in colon cancer HT‐29 cells. Pharmacol Rep. 2012;64(3):745‐750. 22814028 10.1016/s1734-1140(12)70870-8

[55] Walczak K , Langner E , Szalast K , Makuch‐Kocka A , Pożarowski P , Plech T . A tryptophan metabolite, 8‐hydroxyquinaldic acid, exerts antiproliferative and anti‐migratory effects on colorectal cancer cells. Molecules. 2020;25(7):1655. 32260268 10.3390/molecules25071655 PMC7181169

[56] Walczak K , Zurawska M , Kiś J , et al. Kynurenic acid in human renal cell carcinoma: its antiproliferative and antimigrative action on caki‐2 cells. Amino Acids. 2012;43(4):1663‐1670. 22349835 10.1007/s00726-012-1247-5

[57] Walczak K , Turski WA , Rajtar G . Kynurenic acid inhibits colon cancer proliferation in vitro: effects on signaling pathways. Amino Acids. 2014;46(10):2393‐2401. 25012123 10.1007/s00726-014-1790-3 PMC4168223

[58] Abd El‐Fattah EE . IDO/kynurenine pathway in cancer: possible therapeutic approaches. J Transl Med. 2022;20(1):347. 35918736 10.1186/s12967-022-03554-w PMC9344609

[59] Quintero‐Fabián S , Arreola R , Becerril‐Villanueva E , et al. Role of matrix metalloproteinases in angiogenesis and cancer. Front Oncol. 2019;9:1370. 31921634 10.3389/fonc.2019.01370 PMC6915110

[60] Mailankot M , Staniszewska MM , Butler H , et al. Indoleamine 2,3‐dioxygenase overexpression causes kynurenine‐modification of proteins, fiber cell apoptosis and cataract formation in the mouse lens. Lab Invest. 2009;89(5):498‐512. 19308046 10.1038/labinvest.2009.22 PMC2722445

[61] Akhmetova DA , Kozlov VV , Gulyaeva LF . New insight into the role of AhR in lung carcinogenesis. Biochemistry (Mosc). 2022;87(11):1219‐1225. 36509717 10.1134/S0006297922110013

[62] Xue P , Fu J , Zhou Y . The aryl hydrocarbon receptor and tumor immunity. Front Immunol. 2018;9:286. 29487603 10.3389/fimmu.2018.00286 PMC5816799

[63] Yin J , Sheng B , Qiu Y , Yang K , Xiao W , Yang H . Role of AhR in positive regulation of cell proliferation and survival. Cell Prolif. 2016;49(5):554‐560. 27523394 10.1111/cpr.12282 PMC6496171

[64] Marszalek‐Grabska M , Walczak K , Gawel K , et al. Kynurenine emerges from the shadows—current knowledge on its fate and function. Pharmacol Ther. 2021;225:107845. 33831481 10.1016/j.pharmthera.2021.107845

[65] Platten M , Wick W , van den Eynde BJ . Tryptophan catabolism in cancer: beyond IDO and tryptophan depletion. Cancer Res. 2012;72(21):5435‐5440. 23090118 10.1158/0008-5472.CAN-12-0569

[66] Safe S , Lee SO , Jin UH . Role of the aryl hydrocarbon receptor in carcinogenesis and potential as a drug target. Toxicol Sci. 2013;135(1):1‐16. 23771949 10.1093/toxsci/kft128 PMC3748760

[67] Amobi‐McCloud A , Muthuswamy R , Battaglia S , et al. IDO1 expression in ovarian cancer induces PD‐1 in T cells via aryl hydrocarbon receptor activation. Front Immunol. 2021;12:678999. 34025677 10.3389/fimmu.2021.678999 PMC8136272

[68] Anzai H , Yoshimoto S , Okamura K , Hiraki A , Hashimoto S . IDO1‐mediated Trp‐kynurenine‐AhR signal activation induces stemness and tumor dormancy in oral squamous cell carcinomas. Oral Sci Int. 2022;19(1):31‐43.

[69] Atene CG , Fiorcari S , Mesini N , et al. Indoleamine 2,3‐dioxygenase 1 mediates survival signals in chronic lymphocytic leukemia via kynurenine/aryl hydrocarbon receptor‐mediated mcl1 modulation. Front Immunol. 2022;13:832263. 35371054 10.3389/fimmu.2022.832263 PMC8971515

[70] Contador‐Troca M , Alvarez‐Barrientos A , Barrasa E , et al. The dioxin receptor has tumor suppressor activity in melanoma growth and metastasis. Carcinogenesis. 2013;34(12):2683‐2693. 23843039 10.1093/carcin/bgt248

[71] Serio CD , Cozzi A , Angeli I , et al. Kynurenic acid inhibits the release of the neurotrophic fibroblast growth factor (FGF)‐1 and enhances proliferation of glia cells, in vitro. Cell Mol Neurobiol. 2005;25(6):981‐993. 16392031 10.1007/s10571-005-8469-y PMC11529496

[72] Pires AS , Sundaram G , Heng B , Krishnamurthy S , Brew BJ , Guillemin GJ . Recent advances in clinical trials targeting the kynurenine pathway. Pharmacol Ther. 2022;236:108055. 34929198 10.1016/j.pharmthera.2021.108055

[73] Cheong JE , Sun L . Targeting the IDO1/TDO2‐KYN‐AhR pathway for cancer immunotherapy—challenges and opportunities. Trends Pharmacol Sci. 2018;39(3):307‐325. 29254698 10.1016/j.tips.2017.11.007

